# Artificial Intelligence in Medicine and Radiation Oncology

**DOI:** 10.7759/cureus.2475

**Published:** 2018-04-13

**Authors:** Vincent Weidlich, Georg A. Weidlich

**Affiliations:** 1 Kingston Business School, Kingston University; 2 Radiation Oncology, National Medical Physics and Dosimetry Comp., Inc

**Keywords:** artificial intelligence, radiation oncology, process efficiency, quality improvement, error prevention, process optimization, error analysis, machine learning, big data

## Abstract

Artifical Intelligence (AI) was reviewed with a focus on its potential applicability to radiation oncology. The improvement of process efficiencies and the prevention of errors were found to be the most significant contributions of AI to radiation oncology. It was found that the prevention of errors is most effective when data transfer processes were automated and operational decisions were based on logical or learned evaluations by the system. It was concluded that AI could greatly improve the efficiency and accuracy of radiation oncology operations.

## Introduction and background

According to Winston Churchill, Alan Turing shortened World War II by two years. He developed the ‘Turing machine’ that was instrumental in breaking the German enigma code, thereby allowing the British secret service to obtain critical strategic information from Nazi enemy forces. Turing also created the first chess computer program and designed the programming of the world’s first commercial computer. He is considered to be the pioneer of artificial intelligence (AI), enabling a number of subsequent critical developments in computer science and machine learning that would not have been possible without Turing’s contributions.

AI has been a growing research field in the past decade, affecting the fabric of employment and the way we think about our life in the workplace. Embracing AI will improve efficiency and the processes historically performed by humans. Certain repetitive jobs have been replaced by AI because its application provides optimized, efficient results in multitasking environments and many advancements have been made in the development and mapping of machine learning. Applied AI and machine learning provide the more precise and faster execution of tasks and can be employed in multiple areas simultaneously. With computing power and technology growing at a fast pace, AI, along with robotics, is at the cutting edge of the development of medicine.

Radiation oncology has several weak points, which are mainly found in the transcription processes of transferring information from one process of radiation oncology to the next. A potential for error is created at these critical junctions due to human involvement and error. This effect can be minimized or eliminated by implementing AI. The radiation oncology teams, such as radiation oncologists, physicists, dosimetrists, and therapists, have developed methods and models that can be internalized and improved upon by A.I. This will be accomplished by using methods and algorithms, applying specific rules, and incrementally improving rules and processes.

## Review

AI, as well as robotics, has been progressing for the past 40 years in areas ranging from automation in manufacturing, where the speed and efficiency of assembly lines were greatly improved, to data processing facilities, and many other commercial organizations. This led to robotics advancements, which, however, has largely bypassed the medical industry, with the exception of certain robotic devices, such as the CyberKnife (Accuray, Sunnyvale, California, USA) [1], ocular microsurgery devices, and the da Vinci robotic microsurgery [2].

Many organizations started to implement AI and have replaced low to mid-level skilled employees with automated processes based on machine learning. In the hospital environment, AI will provide improvements in the areas of general operations, such as patient admissions and inpatient management, where operational efficiencies will be optimized. Furthermore, physician and surgery schedules will be managed by AI, avoiding unnecessary downtime and considering all logical requirements and boundary conditions that exist in similar, complex operations.

For intensive care units, patient monitoring devices, diagnoses, surgery equipment utilization, predictive precautions, as well as vital patient information, trends will be identified and predictive algorithms developed, which, in turn, will be used to avoid future undesired clinical outcomes or staff shortages. AI will assist in patient intake, triage efforts, determining appropriate medication and dosage for patients, notifying care personnel, and keeping logs, based on patient symptoms and vital signs.

**Figure 1:**
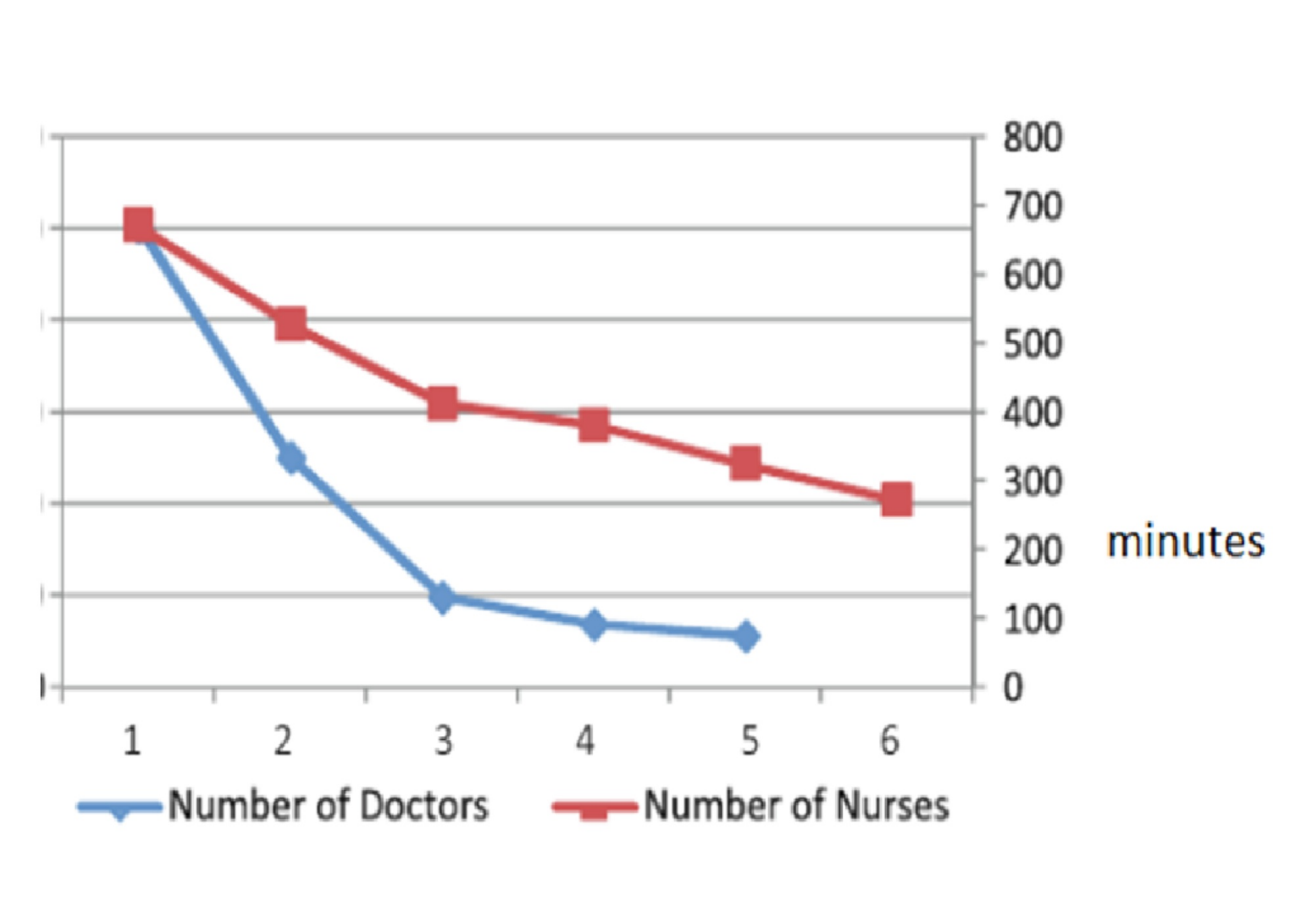
Change in the number of doctors and nurses impacting the length of a patient's hospital stay in minutes

**Figure 2:**
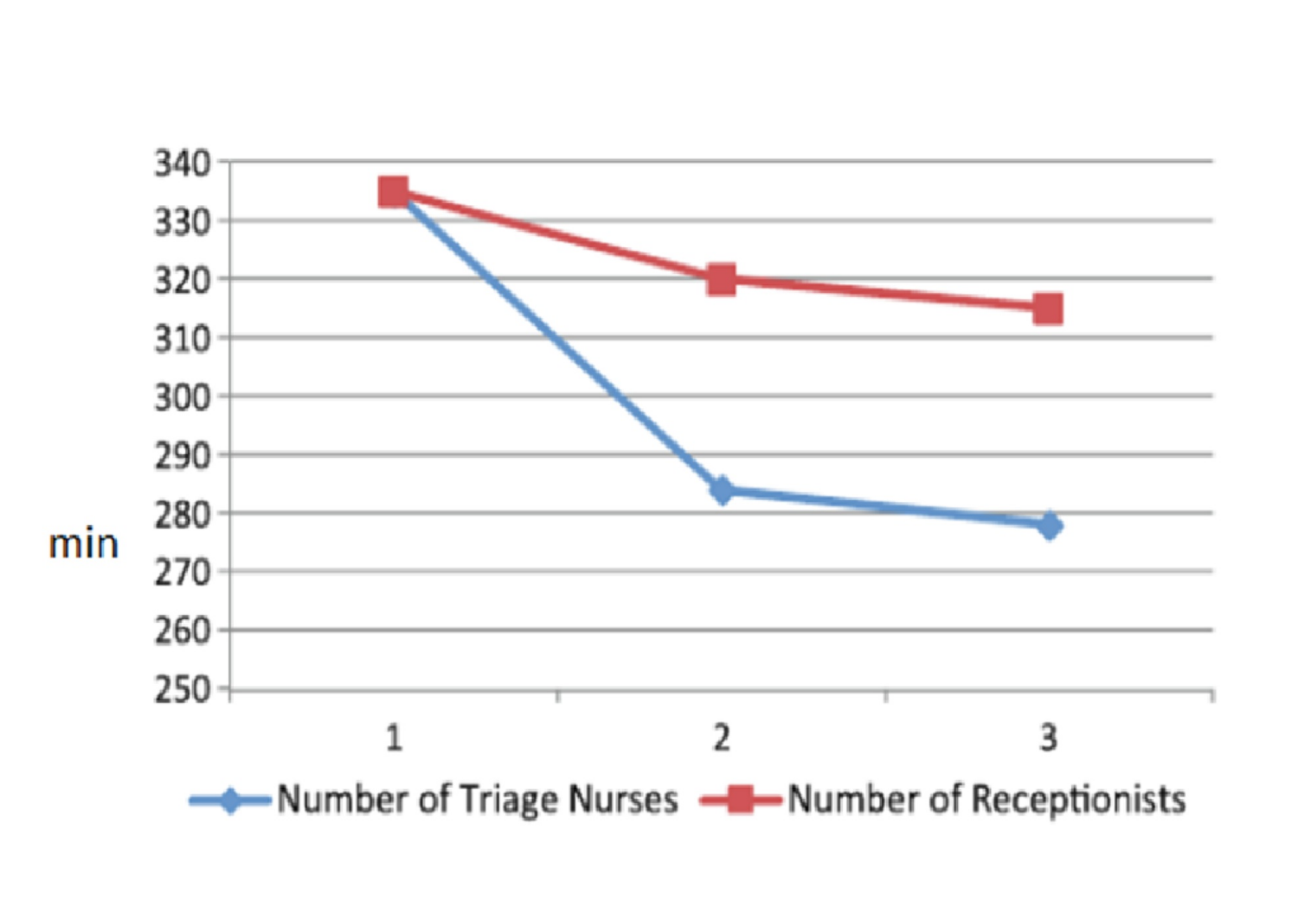
Change in the number of triage nurses impacting the average length of a patient's hospital stay in minutes

**Figure 3:**
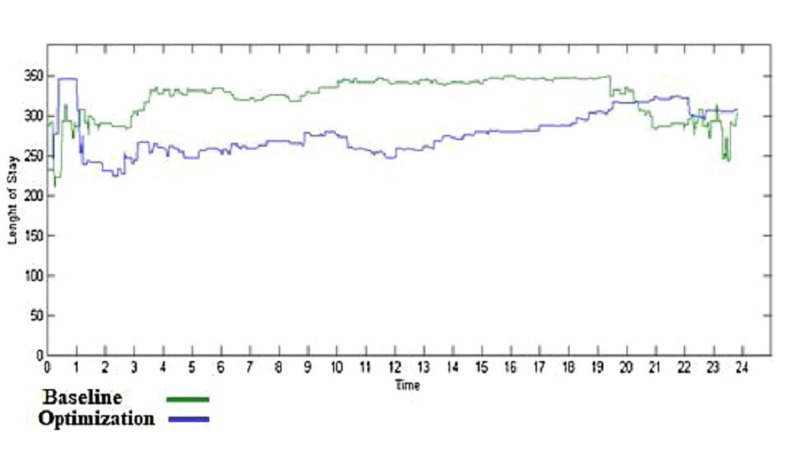
Comparison of length of hospital stay in minutes in baseline resource planning and genetic algorithm results

Emergency rooms, with large amounts of patients visiting every day, will be made much more efficient by the use of AI. For example, a system that records vital signs and poses patient queries, being connected to the hospital server, can decrease waiting time and help triage more serious emergencies to be treated earlier. In a report by Yousefi et al. [3], AI made a significant impact by changing the number and assignments of doctors and nurses to minimize the length of a patient’s hospital stay. This was in part accomplished by changing the number and work-shift length of the care personnel. The impact of nurses, doctors (Figure 1), and receptionists (Figure 2) are shown below. It was concluded that the length of the hospital stay was shortened by increasing staff levels but beyond a certain increase, no further benefit was achieved. The length of a patient’s stay was decreased by the implementation of a computer algorithm in baseline resource planning, as shown in Figure 3. Only after reaching 20 hours of operations was the patient's length of stay not significantly decreased by the optimization algorithm in an inpatient facility. Translated to an outpatient radiation oncology operation, with shorter patient visits and the need for optimized patient flow, it is anticipated that the positive impact of such an algorithm will be most effective for the rapid sequence of patient check-in, queuing, and setups for treatment, imaging, and treating.

When examining a patient, AI will assist doctors in making a faster and more precise diagnosis. AI can run in ghost mode to analyze and keep records and scores of successful predictions. A view of this record will show empirical and statistical evidence without having to reveal the structure or program of the AI. Patients and users might have unrealistic expectations of any newly installed AI, but this can be avoided by informing and briefing. Liability will be placed on the utilizing institution, which can use legal action on the manufacturer, if necessary.

In recent years, the application of machine learning to cancer disease modeling has become a fast-growing trend in research due to the ability of AI to decipher complex datasets for both disease recurrence and patient survival [4]. A study by Francis et al. [5], analyzing post-op data from colorectal cancer surgery patients with a neural network, yielded results compliant with standard regression methods and identified definitive reasons for readmission within the first 30 days after surgery.

A recent study by Chen et al. [6] used the random forest, radial basis function, and neural network algorithms as base learners and AdaBoostM1, Real AdaBoost, and MultiBoostAB as ensemble techniques to address the problem of the low efficiency and slow speed of optimizations. Cancer survivability prediction models based three variants of the AdaBoost algorithm to extend the application range of ensemble methods. The concurrent use of thermography and artificial neural networks (ANN) for the diagnosis of breast cancer was used in a study by Ng et al. [7]. An intelligent breast thermography-neural network will be able to give an accurate diagnosis of breast cancer and can make a positive impact on breast disease detection.

In an interdisciplinary study of medical injury and malpractice litigation by Brennan et al. [8], adverse events were observed in over 3.7% of hospitalized patients with high confidence and over one-quarter of the adverse events were found to be caused by negligence; 2.6% caused permanently disabling injuries and 13.6% of those led to death. Severe injuries increased in probability due to negligence. Of the 2,671,863 patients discharged from New York hospitals in one calendar year, 98,609 were adverse events and 27,179 were adverse events involving negligence. Building on these findings, AI will be used to identify probabilities for such events, design counter-active measures, and improve the measures according to the learned experiences.

Precision medicine relies on an increasing amount of heterogeneous data. Advancements in radiation oncology, through the use of computed tomography (CT) scan, dosimetry, and imaging performed before the delivery of each treatment fraction, have generated a considerable flow of data that needs to be integrated [9]. At the same time, electronic health records now provide phenotypic profiles of large cohorts of patients that could be correlated to this information. The potential uses of machine learning methods, such as support vector machines, artificial neural networks, and deep learning, are to be considered.

AI will ensure the accurate transfer of information from hospital servers to the treatment planning system, the record-and-verify system, and the treatment delivery system. Physicists and treatment planning teams will assist in providing rules and methods for the program, as well as alternative ways to improve its accuracy. Computers can treat more precisely. Many clinical incidents occur in radiation treatment due to human error and equipment failures [10]. A systematic approach to collecting and processing incidents' data is required to manage patient risks. Past successful approaches have included the creation of committed customer focus groups, possibly entering into a non-disclosure agreement, in which errors and misadministrations can be freely shared, yet remain protected from public scrutiny, and group corrective actions and monitoring processes can be put into place. This effort is expected to significantly accelerate the user community learning from adverse events and overcome the potential for error. This effort is not recognized by existing billing codes, but it is suggested here that fiscal recognition by the legislature of a global system clinical quality assurance effort should be pursued.

The Patient Safety and Quality Improvement Act of 2005 established essential legal protections in the United States to allow for the collection and analysis of medical incidents nationwide [11]. The American Society for Radiation Oncology and the American Association of Physicists in Medicine established the radiation oncology incident learning system (RO-ILS). Presently, RO-ILS is actively collecting, analyzing, and reporting patient safety events. Learned experiences from the collected data are used to design systems not only optimized for efficiency but also for error minimization and elimination.

Error resolutions will be uploaded to the server so that all connected AI radiation therapy programs will be updated to avoid potential future errors. By optimizing processes, efficiencies, treatment consistency, and possible after-care issues will improve as well. Radiation therapy devices already have self-calibrating mechanisms, and AI can interface with these mechanisms, to provide consistent operation and treatment delivery. In addition, biases in any areas of operation will be eliminated. By using the Bayesian inference, Monte Carlo methods [12], Markov chain, and Markov chain Monte Carlo (MCMC) algorithms, as well as deep learning, data mining, predictive analytics, and genomics in health care, the accuracy and outcome of diagnosis, prognosis, and treatment of patients will be greatly improved.

The American Association of Physicists in Medicine assembled a panel of experts and tasked them with developing consensus recommendations in five key areas: definitions, process maps, severity scales, causality taxonomy, and data elements [13]. Recommendations were made for these areas, which include a 10-level medical severity scale designed to reflect the observed or estimated harm to a patient, a radiation oncology-specific root causes table to facilitate and regularize root-cause analyses, and recommendations for data elements and structures to aid in the development of electronic databases. The automation of this process will lead to a self-adjusting process of learning from the documented mistakes that will minimize future errors, misadministration, and sentinel events.

AI will learn optimized processes to improve treatment planning and post-treatment evaluation. It will build on these processes to further improve efficiency and minimize errors. Treatment planning can be improved by allowing the optimization of processes and dosimetric results during the planning process. AI will verify transcriptions at all critical junctions of the radiation oncology process, such as prescription-to-plan, plan-to-record-and-verify, and to the treatment unit.

## Conclusions

Applied AI and machine learning provide more precise and faster executions of tasks and can be employed in multiple areas simultaneously. With computing power and technology growing at a fast pace, AI, along with robotics, is at the cutting edge of the development of medicine. AI will learn optimized processes to improve treatment planning and post-treatment evaluation. It will build on these processes to further improve efficiency and minimize errors. AI will also improve treatment efficiency, reduce transcription errors, steepen the learning curve for new programs, automate the radiation therapy process by the generation of templates, identify problematic trends, correct for potential errors, monitor corrections, and standardize the process in optimized ways. In addition, AI can be used to verify treatment plans and treatment delivery by comparing them to previous and programmed templates. We are planning to work with several hospitals, implementing AI to improve efficiency and accuracy in radiation oncology. AI will govern plan generation and treatment delivery, as well as verification of treatment. In addition, AI will replace repetitive tasks in dosimetry, allowing the team members to focus on creativity and innovation. It can be concluded that the prevention of errors is most effective when data transfer processes were automated and that AI can greatly improve the efficiency and accuracy of radiation oncology operations. The authors recognize the slow and ineffective past efforts to implement AI in medicine and radiation oncology, which was mainly caused by an institution's prospect of potential legal exposure based on the discoverable self-documentation of errors and misadministrations. To allow AI to gain traction in improving and optimizing radiation oncology processes, it is recognized that legislature will need to be put in place that will allow the legal protection of institutions that are committed to the rigorous process of error recording and documenting institutional efforts to minimize misadministrations. Such a minimization of a hospital's liability is expected to set the stage for a systematic AI-supported approach to error prevention and process automation. Future research will include virtual reality applications to provide a more relevant perspective for the end user, by allowing the visualization of a treatment plan from a patient’s internal vantage point.
